# 2-(4-Methyl­phen­yl)-1*H*-anthraceno[1,2-*d*]imidazole-6,11-dione: a fluorescent chemosensor

**DOI:** 10.1107/S1600536809013634

**Published:** 2009-04-18

**Authors:** Tiago T. Guimarães, Eufrânio N. Da Silva Júnior, Carlos Eduardo M. Carvalho, Carlos A. De Simone, Antonio V. Pinto

**Affiliations:** a Núcleo de Pesquisas em Produtos Naturais, Universidade Federal do Rio de Janeiro, 21944-971 Rio de Janeiro, RJ, Brazil; b Instituto de Química, Universidade de Brasília, 70910-970 Brasília, DF, Brazil; c Universidade Estadual da Zona Oeste (UEZO), 23070-200 Rio de Janeiro, RJ, Brazil; d Instituto de Física de São Carlos, Universidade de São Paulo – USP, 13560-970 São Carlos, SP, Brazil

## Abstract

In the title compound, C_22_H_14_N_2_O_2_, the five rings of the mol­ecule are not coplanar. There is a significant twist between the four fused rings, which have a slightly arched conformation, and the pendant aromatic ring, as seen in the dihedral angle of 13.16 (8)° between the anthraquinonic ring system and the pendant aromatic ring plane.

## Related literature

For general background on organic fluoro­phores, see: Czarnik (1994 ▶); Friend *et al.* (1999 ▶); Joux & Lebaron (2000 ▶); Kasten (1999 ▶); Soukos *et al.* (2000 ▶); Zhu *et al.* (2008 ▶). For related structures and applications, see: Peng *et al.* (2005 ▶); Boiocchi *et al.* (2004 ▶); Yoshida *et al.* (2002 ▶).
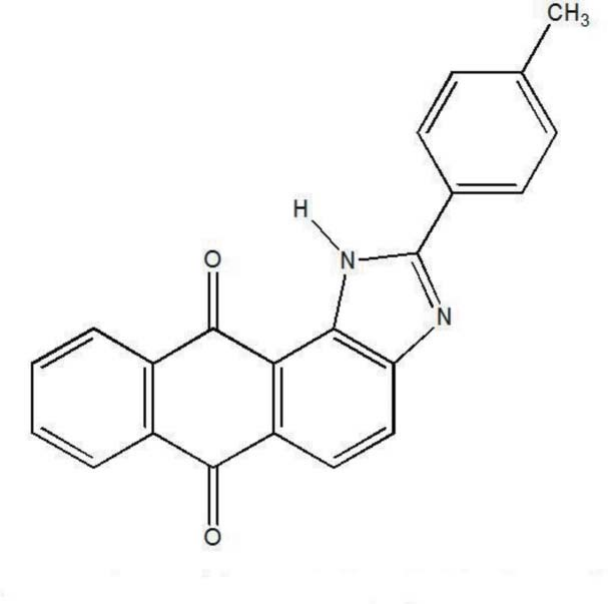


## Experimental

### 

#### Crystal data

C_22_H_14_N_2_O_2_
*M* *_r_* = 338.35
Orthorhombic, 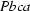

*a* = 7.3850 (10) Å
*b* = 14.0730 (4) Å
*c* = 30.5630 (9) Å
*V* = 3176.4 (4) Å^3^
*Z* = 8
Mo *K*α radiation
μ = 0.09 mm^−1^
*T* = 295 K
0.14 × 0.14 × 0.07 mm

#### Data collection

Nonius KappaCCD diffractometer
Absorption correction: none
20847 measured reflections
3643 independent reflections
2282 reflections with *I* > 2σ(*I*)
*R*_int_ = 0.066

#### Refinement

*R*[*F* ^2^ > 2σ(*F* ^2^)] = 0.060
*wR*(*F* ^2^) = 0.155
*S* = 1.05
3643 reflections
235 parameters
H-atom parameters constrained
Δρ_max_ = 0.18 e Å^−3^
Δρ_min_ = −0.20 e Å^−3^

### 

Data collection: *COLLECT* (Nonius, 2000 ▶); cell refinement: *SCALEPACK* (Otwinowski & Minor, 1997 ▶); data reduction: *DENZO* (Otwinowski & Minor, 1997 ▶) and *SCALEPACK*; program(s) used to solve structure: *SHELXS97* (Sheldrick, 2008 ▶); program(s) used to refine structure: *SHELXL97* (Sheldrick, 2008 ▶); molecular graphics: *ORTEP-3 for Windows* (Farrugia, 1997 ▶); software used to prepare material for publication: *WinGX* (Farrugia, 1999 ▶).

## Supplementary Material

Crystal structure: contains datablocks I, global. DOI: 10.1107/S1600536809013634/tk2418sup1.cif

Structure factors: contains datablocks I. DOI: 10.1107/S1600536809013634/tk2418Isup2.hkl

Additional supplementary materials:  crystallographic information; 3D view; checkCIF report

## Figures and Tables

**Fig. 1.**
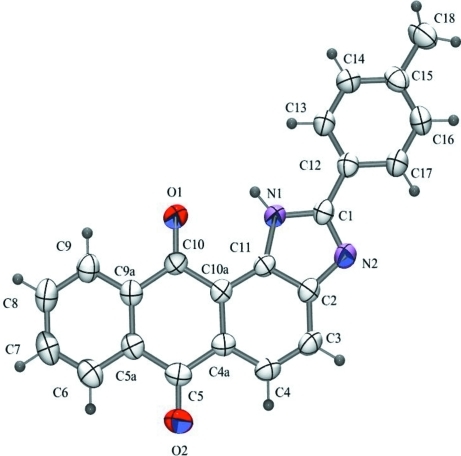
Projection of (I), showing the atom labelling with 50% probability displacement ellipsoids.

## supplementary crystallographic information

### Comment

Recently, much attention has been devoted to the study of organic fluorophores because of their potential use in analytical chemistry (Czarnik, 1994), optoeletronics (Friend *et al.*, 1999), dye technologies (Joux & Lebaron, 1988; Kasten, 1999), forensic chemistry (Soukos *et al.*, 2000), and in pharmaceutical analysis evaluations (Zhu *et al.*, 2008). In addition, specific and sensitive chemosensors for anions are of great importance in environmental science (Peng *et al.*, 2005). The N—H···F interaction is already well known (Boiocchi *et al.*, 2004) and can be exploited in the development of new molecules to function as fluorescent probes for fluoride. For instance, one such class of probe are the anthraimidazolic-derived quinones that can be deprotonated in the presence of anions to enhance their natural fluorescence by a supposed mechanism of photo-induced electron transfer (PET) or *via* a bathochromic shift of the absorption bands promoted by charge transfer (CT) (Peng *et al.*, 2005). Although many photophysical properties of fluorophores are well known in solution, only a few are known in solid-state (Yoshida *et al.*, 2002). In this paper we report the molecular structure of the 2-*p*-tolyl-1*H*-anthra[1,2-*d*]imidazole-6,11-dione, (I), a fluorescent probe synthesized in our laboratory.

In (I), the rings are not co-planar (Fig. 1). The anthraquinonic ring is almost planar with the greatest deviation from the least-squares plane of 0.102 (2) Å being exhibited by atom C7. The dihedral angle between the anthraquinonic ring [C2—C11] and the benzene ring [C12—C17] planes is 13.16 (8)°.

### Experimental

To an acetic acid solution (25 ml)  of the 1,2-diaminoanthraquinone (238 mg, 1 mmol),  *p*-methyl-benzaldehyde (132 mg, 1.1 mmol) and  sodium acetate (107 mg, 1.3 mmol) were added. The mixture was left under agitation and reflux for 30 min. The reaction was leaked into cold water (50 ml) which precipitated a yellow solid that was filtered under vacuum. The new anthraimidazole derivate (I) was purified by column chromatography over silica-gel, using a dichloromethane/ethyl acetate (5:1) mixture as eluent and obtained as yellow crystals in 69.5% yield (235 mg, 0.70 mmol); m.p. 522 K. ^1^H NMR (300 MHz, CDCl_3_) δ: 8.33–8.30 (m, 1H); 8.27–8.24 (m, 1H); 8.20 (d, *J* = 8.79 Hz, 1H); 8.08 (d, *J* = 7.91 Hz, 2H); 8.03 (d, *J* = 8.79 Hz, 1H); 7.83–7.75 (m, 2H); 7.37 (d, *J* = 7.91 Hz, 2H); 2.46 p.p.m. (s, 3H); N—H not obs. ^13^C NMR (300 MHz, CDCl_3_): δ: 21.8, 117.82, 121.87, 125.40, 125.74, 126.37, 126.79, 126.91, 127.48, 128.35, 129.91, 129.91, 133.14, 133.23, 133.63, 133.94, 134.29, 141.94, 149.53, 156.78, 183.1, 183.1 p.p.m.

### Refinement

H atoms were located on stereochemical grounds and refined with fixed geometry, each riding on a carrier atom, with C—H = 0.93 - 0.98 Å and U_iso_ = 1.5 (for methyl-H) and 1.2 (other H atoms) U_eq_(carrier atom).

### Crystal data

**Table:**

C_22_H_14_N_2_O_2_	*F*(000) = 1408
*M**_r_* = 338.35	*D*_x_ = 1.415 Mg m^−^^3^
Orthorhombic, *P**b**c**a*	Mo *K*α radiation, λ = 0.71073 Å
Hall symbol: -P 2ac 2ab	Cell parameters from 14527 reflections
*a* = 7.385 (1) Å	θ = 2.9–27.5°
*b* = 14.0730 (4) Å	µ = 0.09 mm^−^^1^
*c* = 30.5630 (9) Å	*T* = 295 K
*V* = 3176.4 (4) Å^3^	Prism, yellow
*Z* = 8	0.14 × 0.14 × 0.07 mm

### Data collection

**Table:**

Nonius KappaCCD diffractometer	2282 reflections with *I* > 2σ(*I*)
Radiation source: Enraf–Nonius FR590	*R*_int_ = 0.066
horizonally mounted graphite crystal	θ_max_ = 27.5°, θ_min_ = 3.0°
Detector resolution: 9 pixels mm^-1^	*h* = −9→7
CCD rotation images, thick slices scans	*k* = −14→18
20847 measured reflections	*l* = −39→38
3643 independent reflections	

### Refinement

**Table:**

Refinement on *F*^2^	Primary atom site location: structure-invariant direct methods
Least-squares matrix: full	Secondary atom site location: difference Fourier map
*R*[*F*^2^ > 2σ(*F*^2^)] = 0.060	Hydrogen site location: inferred from neighbouring sites
*wR*(*F*^2^) = 0.155	H-atom parameters constrained
*S* = 1.05	*w* = 1/[σ^2^(*F*_o_^2^) + (0.0589*P*)^2^ + 1.2992*P*] where *P* = (*F*_o_^2^ + 2*F*_c_^2^)/3
3643 reflections	(Δ/σ)_max_ < 0.001
235 parameters	Δρ_max_ = 0.18 e Å^−^^3^
0 restraints	Δρ_min_ = −0.20 e Å^−^^3^

### Special details

**Table:**

Geometry. All e.s.d.'s (except the e.s.d. in the dihedral angle between two l.s. planes) are estimated using the full covariance matrix. The cell e.s.d.'s are taken into account individually in the estimation of e.s.d.'s in distances, angles and torsion angles; correlations between e.s.d.'s in cell parameters are only used when they are defined by crystal symmetry. An approximate (isotropic) treatment of cell e.s.d.'s is used for estimating e.s.d.'s involving l.s. planes.
Refinement. Refinement of *F*^2^ against ALL reflections. The weighted *R*-factor *wR* and goodness of fit *S* are based on *F*^2^, conventional *R*-factors *R* are based on *F*, with *F* set to zero for negative *F*^2^. The threshold expression of *F*^2^ > σ(*F*^2^) is used only for calculating *R*-factors(gt) *etc*. and is not relevant to the choice of reflections for refinement. *R*-factors based on *F*^2^ are statistically about twice as large as those based on *F*, and *R*- factors based on ALL data will be even larger.

### Fractional atomic coordinates and isotropic or equivalent isotropic displacement parameters (Å^2^)

**Table:**

	*x*	*y*	*z*	*U*_iso_*/*U*_eq_	
O1	0.7524 (2)	0.58882 (10)	0.24672 (5)	0.0537 (4)	
O2	0.4748 (2)	0.25831 (11)	0.30424 (6)	0.0632 (5)	
N1	0.6678 (2)	0.53709 (11)	0.16053 (6)	0.0413 (4)	
H1N	0.7063	0.5904	0.1706	0.050*	
N2	0.5832 (2)	0.42641 (11)	0.11186 (6)	0.0454 (4)	
C1	0.6423 (3)	0.51458 (14)	0.11723 (7)	0.0420 (5)	
C2	0.5674 (3)	0.39056 (14)	0.15381 (7)	0.0420 (5)	
C3	0.5055 (3)	0.30226 (14)	0.16831 (8)	0.0480 (5)	
H3	0.4709	0.2556	0.1484	0.058*	
C4	0.4967 (3)	0.28566 (14)	0.21264 (8)	0.0476 (5)	
H4	0.4546	0.2272	0.2225	0.057*	
C4A	0.5494 (3)	0.35426 (13)	0.24325 (7)	0.0406 (5)	
C5	0.5310 (3)	0.33481 (15)	0.29076 (8)	0.0454 (5)	
C5A	0.5811 (3)	0.41206 (14)	0.32185 (7)	0.0440 (5)	
C6	0.5545 (3)	0.39803 (18)	0.36653 (8)	0.0567 (6)	
H6	0.5056	0.3412	0.3766	0.068*	
C7	0.6007 (3)	0.46845 (19)	0.39578 (8)	0.0632 (7)	
H7	0.5787	0.4597	0.4255	0.076*	
C8	0.6794 (3)	0.55197 (18)	0.38151 (8)	0.0621 (7)	
H8	0.7133	0.5982	0.4016	0.075*	
C9	0.7077 (3)	0.56666 (16)	0.33750 (8)	0.0506 (6)	
H9	0.7609	0.6228	0.3279	0.061*	
C9A	0.6566 (3)	0.49753 (14)	0.30745 (7)	0.0420 (5)	
C10	0.6808 (3)	0.51561 (14)	0.26033 (7)	0.0399 (5)	
C10A	0.6161 (3)	0.44294 (13)	0.22942 (7)	0.0380 (5)	
C11	0.6208 (3)	0.45918 (13)	0.18450 (7)	0.0381 (5)	
C12	0.6719 (3)	0.58162 (14)	0.08136 (7)	0.0428 (5)	
C13	0.7054 (3)	0.67704 (15)	0.08847 (8)	0.0504 (6)	
H13	0.7151	0.6997	0.1170	0.060*	
C14	0.7245 (3)	0.73941 (16)	0.05371 (8)	0.0558 (6)	
H14	0.7481	0.8032	0.0593	0.067*	
C15	0.7090 (3)	0.70857 (17)	0.01088 (8)	0.0526 (6)	
C16	0.6790 (3)	0.61297 (17)	0.00396 (8)	0.0595 (6)	
H16	0.6702	0.5904	−0.0246	0.071*	
C17	0.6617 (3)	0.55016 (16)	0.03836 (8)	0.0560 (6)	
H17	0.6429	0.4860	0.0327	0.067*	
C18	0.7200 (4)	0.7767 (2)	−0.02702 (9)	0.0722 (8)	
H18A	0.8401	0.8026	−0.0287	0.108*	
H18B	0.6345	0.8273	−0.0228	0.108*	
H18C	0.6926	0.7437	−0.0537	0.108*	

### Atomic displacement parameters (Å^2^)

**Table:**

	*U*^11^	*U*^22^	*U*^33^	*U*^12^	*U*^13^	*U*^23^
O1	0.0679 (10)	0.0417 (8)	0.0517 (10)	−0.0081 (7)	−0.0037 (8)	−0.0011 (7)
O2	0.0776 (11)	0.0491 (9)	0.0630 (11)	−0.0064 (8)	0.0067 (9)	0.0101 (8)
N1	0.0463 (9)	0.0352 (9)	0.0424 (11)	−0.0031 (7)	−0.0018 (8)	−0.0024 (8)
N2	0.0500 (10)	0.0411 (10)	0.0452 (11)	0.0008 (8)	0.0006 (8)	−0.0064 (8)
C1	0.0414 (11)	0.0418 (11)	0.0427 (13)	0.0036 (9)	−0.0024 (9)	−0.0058 (10)
C2	0.0402 (10)	0.0403 (11)	0.0456 (12)	0.0030 (9)	−0.0003 (9)	−0.0045 (10)
C3	0.0529 (13)	0.0362 (11)	0.0550 (15)	−0.0020 (9)	0.0011 (11)	−0.0098 (10)
C4	0.0491 (12)	0.0351 (11)	0.0586 (15)	−0.0019 (9)	0.0037 (11)	−0.0012 (10)
C4A	0.0379 (10)	0.0360 (10)	0.0478 (13)	0.0042 (8)	0.0018 (9)	0.0012 (10)
C5	0.0398 (11)	0.0414 (12)	0.0550 (14)	0.0047 (9)	0.0035 (10)	0.0074 (10)
C5A	0.0382 (11)	0.0488 (12)	0.0452 (13)	0.0075 (9)	−0.0030 (9)	0.0022 (10)
C6	0.0529 (13)	0.0672 (15)	0.0499 (15)	0.0062 (11)	0.0004 (11)	0.0078 (13)
C7	0.0656 (15)	0.0835 (19)	0.0405 (14)	0.0123 (14)	−0.0019 (12)	0.0012 (13)
C8	0.0679 (16)	0.0670 (16)	0.0515 (16)	0.0107 (13)	−0.0098 (12)	−0.0144 (13)
C9	0.0547 (13)	0.0490 (12)	0.0482 (14)	0.0087 (10)	−0.0067 (11)	−0.0057 (11)
C9A	0.0400 (11)	0.0428 (11)	0.0432 (13)	0.0098 (9)	−0.0037 (9)	−0.0006 (10)
C10	0.0391 (10)	0.0338 (10)	0.0468 (13)	0.0044 (9)	−0.0032 (9)	0.0005 (9)
C10A	0.0344 (10)	0.0369 (10)	0.0426 (12)	0.0051 (8)	0.0004 (8)	−0.0027 (9)
C11	0.0369 (10)	0.0341 (10)	0.0435 (12)	0.0013 (8)	−0.0002 (9)	−0.0029 (9)
C12	0.0431 (11)	0.0441 (12)	0.0412 (12)	0.0005 (9)	0.0005 (9)	−0.0028 (10)
C13	0.0598 (14)	0.0479 (13)	0.0434 (13)	−0.0037 (10)	−0.0031 (10)	−0.0052 (10)
C14	0.0656 (15)	0.0490 (13)	0.0526 (15)	−0.0101 (11)	−0.0020 (11)	0.0014 (11)
C15	0.0445 (12)	0.0649 (15)	0.0484 (14)	−0.0049 (11)	0.0024 (10)	0.0066 (12)
C16	0.0709 (16)	0.0690 (16)	0.0387 (13)	−0.0046 (13)	0.0007 (11)	−0.0036 (12)
C17	0.0713 (15)	0.0505 (13)	0.0463 (14)	−0.0040 (11)	0.0021 (11)	−0.0087 (11)
C18	0.0713 (17)	0.0866 (19)	0.0587 (17)	−0.0103 (14)	−0.0009 (13)	0.0207 (15)

### Geometric parameters (Å, °)

**Table:**

O1—C10	1.231 (2)	C7—H7	0.9300
O2—C5	1.225 (2)	C8—C9	1.377 (3)
N1—C11	1.364 (2)	C8—H8	0.9300
N1—C1	1.374 (3)	C9—C9A	1.390 (3)
N1—H1N	0.8600	C9—H9	0.9300
N2—C1	1.325 (2)	C9A—C10	1.473 (3)
N2—C2	1.383 (3)	C10—C10A	1.472 (3)
C1—C12	1.463 (3)	C10A—C11	1.392 (3)
C2—C3	1.396 (3)	C12—C13	1.383 (3)
C2—C11	1.403 (3)	C12—C17	1.389 (3)
C3—C4	1.376 (3)	C13—C14	1.385 (3)
C3—H3	0.9300	C13—H13	0.9300
C4—C4A	1.399 (3)	C14—C15	1.384 (3)
C4—H4	0.9300	C14—H14	0.9300
C4A—C10A	1.407 (3)	C15—C16	1.380 (3)
C4A—C5	1.484 (3)	C15—C18	1.506 (3)
C5—C5A	1.491 (3)	C16—C17	1.380 (3)
C5A—C6	1.393 (3)	C16—H16	0.9300
C5A—C9A	1.397 (3)	C17—H17	0.9300
C6—C7	1.378 (3)	C18—H18A	0.9600
C6—H6	0.9300	C18—H18B	0.9600
C7—C8	1.382 (3)	C18—H18C	0.9600
			
C11—N1—C1	107.29 (16)	C9—C9A—C5A	120.2 (2)
C11—N1—H1N	126.4	C9—C9A—C10	119.47 (19)
C1—N1—H1N	126.4	C5A—C9A—C10	120.34 (19)
C1—N2—C2	104.75 (17)	O1—C10—C10A	120.28 (19)
N2—C1—N1	112.35 (18)	O1—C10—C9A	121.81 (19)
N2—C1—C12	124.07 (19)	C10A—C10—C9A	117.91 (18)
N1—C1—C12	123.55 (18)	C11—C10A—C4A	116.79 (18)
N2—C2—C3	130.29 (19)	C11—C10A—C10	120.72 (18)
N2—C2—C11	110.19 (17)	C4A—C10A—C10	122.48 (19)
C3—C2—C11	119.5 (2)	N1—C11—C10A	131.92 (18)
C4—C3—C2	118.6 (2)	N1—C11—C2	105.41 (18)
C4—C3—H3	120.7	C10A—C11—C2	122.65 (18)
C2—C3—H3	120.7	C13—C12—C17	117.9 (2)
C3—C4—C4A	121.86 (19)	C13—C12—C1	122.37 (19)
C3—C4—H4	119.1	C17—C12—C1	119.70 (19)
C4A—C4—H4	119.1	C12—C13—C14	120.9 (2)
C4—C4A—C10A	120.6 (2)	C12—C13—H13	119.6
C4—C4A—C5	120.09 (18)	C14—C13—H13	119.6
C10A—C4A—C5	119.33 (18)	C15—C14—C13	121.2 (2)
O2—C5—C4A	121.5 (2)	C15—C14—H14	119.4
O2—C5—C5A	120.7 (2)	C13—C14—H14	119.4
C4A—C5—C5A	117.81 (18)	C16—C15—C14	117.6 (2)
C6—C5A—C9A	119.1 (2)	C16—C15—C18	120.8 (2)
C6—C5A—C5	119.1 (2)	C14—C15—C18	121.6 (2)
C9A—C5A—C5	121.8 (2)	C17—C16—C15	121.5 (2)
C7—C6—C5A	119.9 (2)	C17—C16—H16	119.2
C7—C6—H6	120.0	C15—C16—H16	119.2
C5A—C6—H6	120.0	C16—C17—C12	120.8 (2)
C6—C7—C8	120.8 (2)	C16—C17—H17	119.6
C6—C7—H7	119.6	C12—C17—H17	119.6
C8—C7—H7	119.6	C15—C18—H18A	109.5
C9—C8—C7	120.0 (2)	C15—C18—H18B	109.5
C9—C8—H8	120.0	H18A—C18—H18B	109.5
C7—C8—H8	120.0	C15—C18—H18C	109.5
C8—C9—C9A	120.0 (2)	H18A—C18—H18C	109.5
C8—C9—H9	120.0	H18B—C18—H18C	109.5
C9A—C9—H9	120.0		
			
C2—N2—C1—N1	0.8 (2)	C5A—C9A—C10—C10A	2.6 (3)
C2—N2—C1—C12	−177.41 (18)	C4—C4A—C10A—C11	2.0 (3)
C11—N1—C1—N2	−0.6 (2)	C5—C4A—C10A—C11	−176.46 (17)
C11—N1—C1—C12	177.64 (18)	C4—C4A—C10A—C10	−177.16 (18)
C1—N2—C2—C3	177.6 (2)	C5—C4A—C10A—C10	4.4 (3)
C1—N2—C2—C11	−0.7 (2)	O1—C10—C10A—C11	−5.4 (3)
N2—C2—C3—C4	−177.4 (2)	C9A—C10—C10A—C11	174.48 (17)
C11—C2—C3—C4	0.8 (3)	O1—C10—C10A—C4A	173.70 (18)
C2—C3—C4—C4A	−0.6 (3)	C9A—C10—C10A—C4A	−6.4 (3)
C3—C4—C4A—C10A	−0.9 (3)	C1—N1—C11—C10A	−178.3 (2)
C3—C4—C4A—C5	177.60 (18)	C1—N1—C11—C2	0.1 (2)
C4—C4A—C5—O2	2.1 (3)	C4A—C10A—C11—N1	176.36 (19)
C10A—C4A—C5—O2	−179.44 (19)	C10—C10A—C11—N1	−4.5 (3)
C4—C4A—C5—C5A	−177.22 (18)	C4A—C10A—C11—C2	−1.8 (3)
C10A—C4A—C5—C5A	1.2 (3)	C10—C10A—C11—C2	177.34 (17)
O2—C5—C5A—C6	−3.3 (3)	N2—C2—C11—N1	0.4 (2)
C4A—C5—C5A—C6	176.00 (18)	C3—C2—C11—N1	−178.13 (17)
O2—C5—C5A—C9A	175.73 (19)	N2—C2—C11—C10A	179.00 (17)
C4A—C5—C5A—C9A	−5.0 (3)	C3—C2—C11—C10A	0.5 (3)
C9A—C5A—C6—C7	0.7 (3)	N2—C1—C12—C13	169.3 (2)
C5—C5A—C6—C7	179.74 (19)	N1—C1—C12—C13	−8.7 (3)
C5A—C6—C7—C8	−2.3 (3)	N2—C1—C12—C17	−8.8 (3)
C6—C7—C8—C9	1.9 (4)	N1—C1—C12—C17	173.16 (19)
C7—C8—C9—C9A	0.1 (3)	C17—C12—C13—C14	1.2 (3)
C8—C9—C9A—C5A	−1.7 (3)	C1—C12—C13—C14	−177.0 (2)
C8—C9—C9A—C10	177.59 (19)	C12—C13—C14—C15	0.7 (4)
C6—C5A—C9A—C9	1.3 (3)	C13—C14—C15—C16	−1.8 (3)
C5—C5A—C9A—C9	−177.70 (18)	C13—C14—C15—C18	176.8 (2)
C6—C5A—C9A—C10	−178.00 (18)	C14—C15—C16—C17	1.1 (3)
C5—C5A—C9A—C10	3.0 (3)	C18—C15—C16—C17	−177.5 (2)
C9—C9A—C10—O1	3.2 (3)	C15—C16—C17—C12	0.7 (4)
C5A—C9A—C10—O1	−177.51 (18)	C13—C12—C17—C16	−1.9 (3)
C9—C9A—C10—C10A	−176.75 (17)	C1—C12—C17—C16	176.3 (2)
